# COMPARATIVE ANALYSIS OF qPCR MEASUREMENT OF HIV VIRAL LOAD AND ELISA DETECTION OF p24 ANTIGEN AFTER HYPERBARIC OXYGEN EXPOSURE

**DOI:** 10.21010/ajid.v14i2.9

**Published:** 2020-07-31

**Authors:** Retno Budiarti, Kuntaman Kuntaman, Guritno Suryokusumo, Siti Qamariyah Khairunisa

**Affiliations:** 1 Department of Microbiology, Faculty of Medicine, Hang Tuah University, Surabaya, Indonesia; 2 Department of Hyperbaric, Faculty of Medicine, Pembangunan Nasional University, Jakarta, Indonesia; 3 Department of HIV, Institute of Tropical Disease, Airlangga University, Surabaya, Indonesia

**Keywords:** ELISA, HIV-1, hyperbaric oxygen, p24 antigen, PCR

## Abstract

**Background:**

A decrease in the number of viruses or viral nucleic acid components will determine whether a therapy successfully eradicates the virus. Sensitivity and specificity are needed to enable easy, precise and efficient diagnosis and evaluation of therapy. This study examined the sensitivity of quantitative PCR (qPCR) for detecting viral nucleic acids as compared with enzyme-linked immunosorbent assay (ELISA) for detecting the human immunodeficiency virus 1 (HIV-1) p24 antigen after hyperbaric oxygen therapy.

**Materials and Methods:**

In this study, peripheral blood mononuclear cells (PBMCs) from healthy whole blood and inoculated HIV-1/MT4 virus in PBMC cultures were isolated. The cultures were exposed to hyperbaric oxygen at 2.4 ATA with 100% O_2_ for 3 × 30 minutes for 5 days. ELISA and qPCR were used to measure the p24 antigen and HIV-1 mRNA, respectively, in the treatment and control groups.

**Result:**

The amounts of p24 antigen and HIV-1 mRNA were significantly different (p = 0.001, p < α). The two examination methods were significantly different. Hyperbaric oxygen therapy can reduce virus numbers, as observed from the p24 antigen and HIV-1 mRNA levels. The treatment group had significantly lower virus numbers than the control group. HIV-1 mRNA detection is more sensitive than p24 antigen detection.

**Conclusion:**

Both qPCR and ELISA have their advantages, depending on whether the goal is to establish, diagnose or monitor antiretroviral therapy or to evaluate disease progression.

## Introduction

Until now, therapy for HIV infection is not aimed at killing the virus, but at inhibiting the enzymes that play a role in its replication so that the virus cannot increase its number. The remaining viruses will be overcome by the natural immune system and the specific immune response of the host. Following HIV infection, the p24 antigen, which is part of the viral capsid, can be detected in the serum. The p24 protein can be detected in the serum several weeks after infection; the amounts increase due to viral replication in the target cells (McNamara and Collins 2013).

To diagnose or determine the success of therapy, the presence of the virus in the host should be determined. In diagnosis, the aim is to find a virus component that indicates the presence of the virus in the host, as a sign that the patient is infected with the virus. Likewise, in the evaluation of therapy, a decrease in the number of viruses or protein components or in the number of enzymes and viral nucleic acid components can determine the success of a therapy in eradicating the virus (Cameron *et al.*, 2001).

Occasionally, the virus is not detected in the serum because anti-retroviral administration has reduced its amounts. Although, clinically, symptoms improve and viral load decreases, the virus remains in the host. Accordingly, an inspection method with high sensitivity and specificity is needed to suppress false positive or false negative numbers and to enable effective diagnosis and easy, precise and efficient evaluation of therapy (Mogensen *et al.*, 2010).

This study aimed to examine the sensitivity of viral examination by quantitative PCR (qPCR), which detects nucleic acid viruses, as compared to enzyme-linked immunosorbent assay (ELISA), which detects the HIV-1 p24 antigen, after hyperbaric oxygen exposure in peripheral blood mononuclear cells (PBMCs).

## Materials and Methods

This study was conducted in several stages. Briefly, PBMCs were isolated from healthy volunteers. Persons with criteria were aged 18-20 years, physically fit, non-smokers, not suffering from any chronic illness, not alcohol drinkers, and willing to fill out informed consent forms to be volunteers in this study.. This study was carried out after we had received approval from the medical research ethics committee of the Airlangga University medical faculty (No. 296/ECK/KEPK/FKUA/2015). 8 ml of venous blood was collected from each volunteer. The PBMCs were cultured in 5% CO_2_ at 37 °C. Then, the cells were exposed to hyperbaric oxygen at 2.4 absolute pressure (ATA) with 100% O_2_ for 3 × 30 minutes, with 5-minute rest at normal air pressure (21%). Exposure was carried out five times a day for five consecutive days. HIV-1 p24 antigen was measured by ELISA, and viral nucleic acid was measured by qPCR. Lastly, the results of the variables were analysed statistically.

### Preparation of PBMCs

Peripheral blood mononuclear cells (PBMCs) are a population of round nucleated blood cells that includes monocytes, lymphocytes and macrophages, which form the immune system. PBMCs were isolated from whole blood using Ficoll-Histopaque density gradient centrifugation. A total of 8 ml venous blood was obtained from healthy volunteers in EDTA-containing tubes. The blood was carefully placed in Ficoll-Histopaque solution and centrifuged at 1500 rpm for 30 minutes at 4 °C. The mononuclear cell interphase was removed and washed three times in phosphate-buffered saline (PBS) solution (Lefort and Kim 2010).

After the ring layer (containing mononuclear cells) was formed, it was removed with a pipette in an oblique position. The layer was transferred to a new tube and the remaining blood was removed. The PBMCs were washed three times with PBS to remove the remaining Ficoll solution: 12–13 ml PBS was added, and the mixture was shaken and centrifuged at 1300 rpm for 5 minutes at 4 °C.

### Culture of PBMCs

At the last rinse, the red cell sediment was taken and the supernatant was removed. The PBMC pellets were dissolved in 1 ml Roswell Park Memorial Institute (RPMI) 1640 medium + 10% fetal bovine serum (FBS) + 1% sodium bicarbonate, and resuspended until completely dissolved. Then, the mixture was transferred to a 1.5-ml Eppendorf tube and the cells were counted using a haemocytometer. The PBMCs were transferred to a 24-well culture plate, to which 2 ml RPMI 1640 medium and 2 μl of phytohaemagglutinin (PHA) were added. The mixture was incubated in 5% CO_2_ at 37°C for 2–3 days. After 3 days, RPMI 1640 medium was added, and the mixture was transferred to a 1.5-ml tube and centrifuged at 1500 rpm for 5 minutes. The supernatant was removed and diluted with RPMI 1640 medium to a total volume of 15 ml, to which 2 μl interleukin-2 (IL-2) was added. Cell counting was performed until the PBMCs numbered 1 × 10^6^ cells. Then, 2 ml of the mixture was transferred into wells and incubated for 3 days.

Infection was carried out by co-cultivating the PBMCs with up to 1 × 10^6^ cells/μl HIV-1/MT4 virus stocks from the biosafety level 3 (BSL3) laboratory at the Institute of Tropical Disease (ITD) of Airlangga University, Surabaya, Indonesia.

### Hyperbaric Oxygen Exposure

Peripheral blood mononuclear cells (PBMCs) cultures were exposed to hyperbaric oxygen by placement in the animal chamber, where they received 2.4 ATA air with 100% O_2_ for 3×30 minutes every day; exposure was carried out for 5 days.

### ELISA Examination of p24 Antigen

Here, a commercial ELISA kit (RETRO-TEK HIV-1 p24 Antigen ELISA, Zeptometrix, Buffalo, NY, USA; catalogue number 0801111) was used. A 600-μl culture sample plus lysis buffer was placed in a RNAse-free tube and vortexed until dispersed and the cell pellet had been lysed. Then, 560 μl 70% ethanol was added to each sample, and then 700 μl per sample was transferred to a spin cartridge (spin column).

The sample was centrifuged at 11,500 rpm for 1 minute, and the liquid at the bottom of the filter was removed; only the liquid in the filter was used. The two procedures above were repeated until all the sample had been used. Then, the spin cartridge was reinserted into the same collection tube.

Wash buffer (700 μl) was added to the sample and centrifuged at 11,500 rpm for 1 minute at room temperature; the liquid under the filter was discarded, and the cartridge was spun and transferred to a new collection tube. Wash buffer II (500 μl) plus ethanol was added to the spin cartridge and it was centrifuged again at 11,500 rpm for 1 minute at room temperature. The two procedures above were repeated twice. Then, the liquid in the spin cartridge was centrifuged at 11,500 rpm for 1 minute to dry the membrane by binding to the RNA in the filter.

The liquid collected under the filter was removed, and the spin cartridge was inserted in to the recovery tube. RNAse-free water (30–100 μl) was added carefully to the middle of the spin cartridge and not to the filter, which is easily torn, and incubated for 1 minute at room temperature. Then, the cartridge was centrifuged at 11,500 rpm for 2 minutes at room temperature to dissolve the RNA from the membrane into the recovery tube. The RNA obtained was placed in a RNAse-free deposit. The pure RNA obtained was stored at -80°C. Once the RNA had been extracted from the samples, it was reverse-transcribed into complementary DNA (cDNA).

### Reverse Transcription (RT) of RNA to cDNA Via Commercial Kit

Each reaction was carried out in a 20-μl volume, which contained 4 μl 5× iScript reaction mix, 1 μl iScript reverse transcriptase, 7 μl nuclease-free water and 8 μl RNA template. The mixture was centrifuged at 6000 × *g* for 15 seconds, then incubated for 5 minutes at 25°C, 30 minutes at 42°C, 5 minutes at 85°C and held at 4°C in the thermocycler. Subsequently, each RNA sample had been reverse-transcribed to cDNA. Then, the concentration of single-stranded (ss) DNA in each sample was measured using a NanoDrop spectrophotometer.

### qRT–PCR amplification

The cDNA obtained was diluted to 50 ng/μl with nuclease-free water, and used for qRT-PCR in a total volume of 10 μl with the following: 5 μl master mix, 1 μl forward primer, 1 μl reverse primer, 1 μl nuclease-free water and 2 μl sample (DNA template). The sequences of the forward and reverse primers for *GAPDH* (glyceraldehyde-3-phosphate dehydrogenase) were 5′-AGGGCTGCTTTTAACTCTGGT-3′ and 3′-CCCCACTTGATTTTGGAGGGA-5′, respectively. The RT-PCR was run for 40 cycles at an annealing temperature of 58°C. The qRT-PCR was conducted in the Institute Tropical Disease (ITD) Bio Safety Level 3 laboratory at UNAIR.

## Results

### ELISA Detection of HIV-1 p24 Antigen

**Figure 1:**
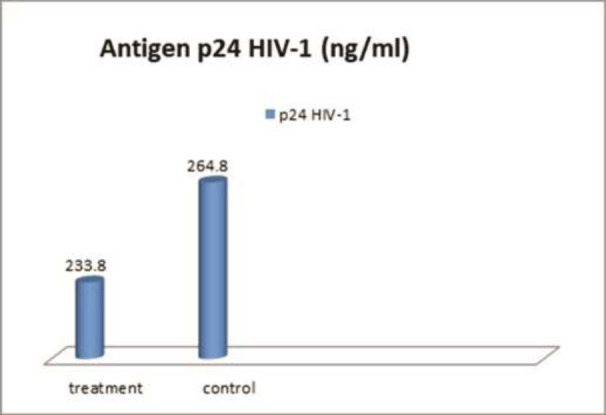
Comparison of the number of p24 HIV-1 antigens.

The HIV virus count was determined by measuring the level of HIV-1 p24 antigen, which is found in the viral capsid by ELISA. The treatment group had a lower p24 antigen concentration than the control group (Fig 1). Kolmogorov-Smirnov analysis showed normal data distribution in each group (p = 0.279; p > α, α = 0.05). The paired *t*-test showed p = 0.017 (p < α, α = 0.05), indicating significant differences in the p24 antigen levels of the treatment and control groups.

### qRT-PCR examination of HIV-1 mRNA

**Figure 2:**
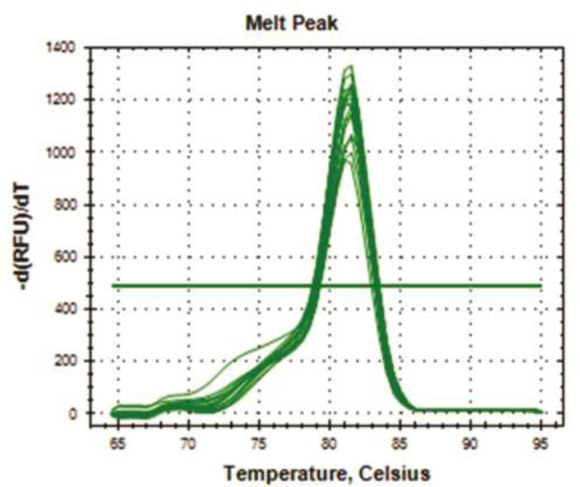
Optimization of housekeeping gene GADPH, shows the same peak, indicating the primer for the gene is specific.

*GAPDH* was used as the housekeeping gene in the qRT-PCR in the present study. A housekeeping gene is used as an internal control (Fig 2). Quantitative gene expression calculation requires correction for the variations in a sample, so it requires normalisation by the housekeeping gene. The suitability of a housekeeping gene is influenced by the type of culture or stimulation, so it can vary between experiments (Löseke *et al.*, 2003).

**Figure 3:**
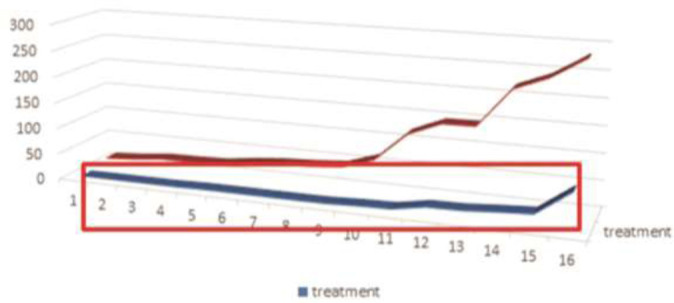
qPCR detection of HIV-1 RNA expression in treatment and control groups (fold) (x: number of samples; y: HIV-1 RNA expression).

*GADPH* was used in this study because it showed stable expression after being optimized; moreover, the *GADPH* gene transcription level was constant at the rate at which HIV-1 mRNA was expressed. There was a significant difference in HIV-1 mRNA between the treatment and untreated groups (Fig 3).

The normal expression of the HIV-1 gene has a value of 1; a positive value > 1 means that there is a more-than-normal increase in gene expression, while a negative value (<0) indicates a decrease in gene expression (downstream regulation).

Kolmogorov-Smirnov testing following RT-PCR showed that the data were normally distributed (p = 0.135; p > α). Paired *t*-test analysis showed that p = 0.001 (p < α). This indicated that there were differences in the expression of the absolute number of HIV-1 RNA between the treatment and control groups.

### Statistical analysis of p24 HIV-1 antigen detection and HIV-1 RNA expression

Statistical analysis was performed using SPSS 20.0. In the treatment group, the Kolmogorov-Smirnov test showed that the data were normally distributed, indicated by α > 0.05. The correlation coefficient was 0.2 (α > 0.05), meaning there was no correlation between the two methods, i.e. ELISA and PCR. The paired *t*-test showed α = 0.000 (α < 0.05), which indicated significant differences between the two methods.

## Discussion

This study shows that qPCR detection of viral nucleic acids is more sensitive than ELISA detection of the HIV-1 p24 antigen for detecting a decrease in HIV-1 numbers after hyperbaric oxygen exposure. HIV-1 replication in PBMC cultures are inhibited after exposure to hyperbaric oxygen. The experimental unit used HIV-infected PBMCs. Inhibition of viral replication was observed by measuring gene expression, protein amounts and virus numbers at the end of the exposure. We decided to use PBMC cultures because no experimental animals other than monkeys have cluster of differentiation 4 (CD4) surface receptors and chemokine receptor 5 (CCR5) and chemokine receptor type 4 (CXCR4) co-receptors, which are required for HIV attachment to host cells.

Enzyme-linked immunosorbent assay (ELISA) measurement of the p24 antigen and qRT-PCR detection of HIV-1 mRNA expression is an indicator of the inhibition of viral replication due to hyperbaric oxygen. The mechanism of hyperbaric oxygen inhibition of the virus is through the expression of genes that produce proteins that can inhibit HIV replication, such as interferon alpha (IFN α) and p21 protein. The statistical analysis showed that the data were normally distributed, so they could be further analysed with the *t*-test.

In homogeneous populations, infected cells produce new virions within 48 hours. Gene expression is detected 18–24 hours after infection. Reverse transcriptase and integrase are present 14.4 hours and 19.3 hours, respectively, after infection. When they begin to express viral proteins, cells die through the cytopathic or lysis effect by cluster of differentiation (CD8) (Holmes *et al.*, 2015). Therefore, in the present study, hyperbaric oxygen was administered on day 3, or 48 hours after viral infection.

### p24 Antigen Levels After Hyperbaric Oxygen Exposure

In accordance with the study purpose of examining the effect of hyperbaric oxygen on decreasing virus numbers, the treatment group had a significantly lower virus number than the control group. Hyperbaric oxygen exposure increases reactive oxygen species (ROS) molecules, which activate the nuclear factor kappa B (NFκB) transcription factor, which then directs the expression of genes that encode antioxidants and natural antivirals.

In the present study, the number of infected cells correlated with that of p24 antigen in the cell cultures, so this examination is a valid measurement of active infection. HIV-1 p24 antigen concentrations in culture supernatant fluids can be observed after day 5 of infection (Yang *et al.*, 2013).

The reactivity properties of ROS allow cells to form antioxidant scavenger molecules that maintain cell homeostasis by increasing the expression of antioxidant genes. ROS also activate transcription factors such as IFN regulatory factor 3 (IRF3) and NFκB to express genes that act as anti-viral and pro-inflammatory cytokines (Olagnier *et al.*, 2014). The p24 antigen can be detected before HIV antibody in newly infected individuals, It is important to remember that not all patients seroconvert will have a detectable p24 antigen, and this antigen may not be found in individuals who have antibodies (Fearon 2005).

The advantage of this examination is that it could detect viruses through the p24 protein in the virus capsid, where only surviving viruses have the capsid component (Yang *et al.*, 2013). The decrease in p24 antigen in the treatment group demonstrates an adaptive response of cells in the form of NFκB activation, which is directed at inhibiting viral replication by increasing p21 protein as a reverse transcriptase inhibitor. The p24 protein is an important structural component of the retrovirus, with an estimated 2000–4000 molecules in each virion.

Although HIV RNA examination is the gold standard, as it is more sensitive than the p24 antigen, the p24 antigen is a good predictor of acute infection and of progression to acquired immune deficiency syndrome (AIDS) or death. The p24 antigen is also useful for anti-retroviral monitoring. p24 is cheaper and easier to examine and does not require difficult pre-treatment procedures.

The principle of p24 antigen detection is the specific binding of antibodies to p24 protein. Unbound components will be washed away when rinsing. The antigen–antibody bonds are detected by markers that have been labelled with horseradish peroxidase or alkaline phosphatase, which causes discoloration. These colour changes are read at specific wavelengths by an ELISA reader. The p24 antigen can be measured in patients with suppressed viremia, and correlate negatively with CD4 T cell concentrations but correlate positively with activated CD8 T cells (Schüpbach *et al.*, 2003).

This examination has three issues: first, the presence of specific antibodies that neutralize p24 so that no immune complex is formed (false negative). Second, the presence of specific antibodies such as rheumatoid factor–like antibodies that facilitate antigen capture and cause over-detection (false positive). Third, low sensitivity when compared with the PCR method.

### qPCR Analysis of HIV-1 RNA

qPCR is a method for detecting the amount of viral RNA. p24 antigen measurement is inferior to RT-PCR for detecting virus particles. Examination of the amount of HIV-1 RNA has become the standard for monitoring the administration of anti-retroviral therapy. The advantage of PCR is that it can detect viral RNA even though virus numbers are too low for ELISA to detect.

A housekeeping gene is a gene that is needed to maintain basic cellular functions and is expressed by all cells under normal and pathological conditions. Such genes must remain constant under different experimental conditions. Housekeeping genes are involved in basic cell metabolism and cell survival processes, and are expressed stably at a constant level. RNA expression analysis using real-time PCR generally uses housekeeping genes as an internal control between samples (Dheda *et al.*, 2004; Kozera and Rapack 2013). According to Barletta (2004), in a study of 55 patients whose viral load in plasma has been pressed for 6 months with antiretroviral therapy, it was found that HIV-1 p24 the antigen detected in this sample was not related to particles that contain viral RNA. suggesting the source of HIV-1 p24 antigen might be located in other compartments of virus expression.

HIV-1 RNA is detected in acute infections, while p24 antigen is positive after 7 days of infection. In this study, the time from viral inoculation to hyperbaric oxygen exposure was approximately 10 days. As many as 5000 virus particles are needed to enable p24 antigen detection. Due to the absence of HIV-specific antibodies, there is no immune complex against the p24 antigen. The difference in characteristics of the p24 antigen and HIV RNA leads to differences in the purpose of examining these markers, for example, for diagnosis, prediction or progression of infection and for anti-retroviral monitoring. A study of 35 sera from patient samples examined by quantitative PCR (qPCR) and ELISA in a sample of HIV-infected patients aged 15-55 years, analyzed for sensitivity with ELISA reactivity which was positively confirmed by qPCR. it was concluded that qPCR was more sensitive and specific than the ELISA method (Aghamirzaei, 2014)

## Conclusion

Hyperbaric oxygen therapy at 2.4 ATA and 100% O_2_ at 3 × 30 minutes for 5 days can inhibit HIV-1 replication in infected PBMCs, which was indicated by a decrease in the HIV-1 p24 antigen and HIV-1 RNA numbers. PCR, which detects the amount of HIV-1 RNA, is more sensitive than ELISA, which detects p24 protein. Both methods are useful depending on the goal, i.e. establishing a diagnosis, monitoring antiretroviral drugs or observing the progression of the disease.

**List of Abbreviation:**
AIDS: : Acquired Immune Deficiency Syndrome
BSL3: : Biosafety Level 3
CCR5: : Surface Receptors and Chemokine Receptor 5
CD4: : Cluster of Differentiation 4
CD8: : Cluster of Differentiation
cDNA: : Complementary DNA
CXCR4: : Chemokine Receptor Type 4
ELISA: : Enzyme-Linked Immunosorbent Assay
FBS: : Fetal Bovine Serum
HIV-1: : Human Immunodeficiency Virus 1
IL-2: : Interleukin-2
IRF3: : IFN Regulatory Factor 3
NFκB: : Nuclear Factor Kappa B
PBMCs: Peripheral Blood Mononuclear Cells; Polymorphonuclear (PMN)
qPCR: : quantitative PCR
ROS: : Reactive Oxygen Species
RPMI: : Roswell Park Memorial Institute.
